# The Effect of EMLA Cream on Patient-Controlled Analgesia with Remifentanil in ESWL Procedure: A Placebo-Controlled Randomized Study

**DOI:** 10.5812/aapm.7790

**Published:** 2013-01-01

**Authors:** Arzu Acar, Elvan Erhan, M. Nuri Deniz, Gulden Ugur

**Affiliations:** 1 Department of Anaesthesiology and Reanimation, School of Medicine, Ege University, Izmir, Turkey

**Keywords:** Lithotripsy, Remifentanil, Analgesia, Patient-Controlled, EMLA

## Abstract

**Background:**

To alleviate stinging pain in the skin entry area and visceral discomfort in patients who are undergoing ESWL.

**Objectives:**

This study was designed to investigate the effectiveness of the EMLA cream in combination with remifentanil patient-controlled analgesia (PCA) in patients undergoing ESWL treatment.

**Patients and Methods:**

Sixty patients were divided into two double-blind randomized groups. Those in the first group were administered 3-5mm of EMLA 5% cream on a marked area; the second group received, as a placebo, a cream with no analgesic effect in the same amount. All patients were administered a remifentanil bolus with a PCA device. Arterial blood pressure, oxygen saturation, and respiratory rate were recorded throughout the procedure; postoperative side effects, agitation, and respiratory depression were measured after. Visual Analogue Scale (VAS) scores were taken preoperatively, perioperatively, directly postoperatively, and 60 minutes subsequent to finishing the procedure.

**Results:**

There were no statistically significant differences in the frequency of PCA demands and delivered boluses or among perioperative VAS. No significant side effects were noted. Patient satisfaction was recorded high in both groups.

**Conclusions:**

EMLA cream offered no advantage over the placebo cream in patients undergoing ESWL with remifentanil PCA.

## 1. Background

Extracorporeal shock wave lithotripsy (ESWL), the most commonly used procedure for the treatment of kidney stones, is painful based on the power of the acoustic shock waves applied (1). Though believed to be multifactorial, the pathogenesis of the pain during ESWL remains to be elucidated. The cutaneous superficial skin nociceptors and visceral nociceptors such as periosteal, pleural, peritoneal and/or musculoskeletal pain receptors are held responsible for the pain (2, 3). Other imperative factors include individual differences, the type of lithotripter, site and size of the stones, and pressure of shock waves (2, 4). During ESWL, general anaesthesia, regional anaesthesia, intravenous anaesthesia or analgesia and sedation can be performed (5, 6). For this purpose, several studies using opioids such as fentanyl, alfentanil, sufentanil, and remifentanil have been conducted (7, 8). Since 1986, various studies have been reported on the use of infiltrative or topical local anesthetics for analgesic purposes. The use of local anesthetics during ESWL has been demonstrated to be effective in achieving analgesia (9, 10).

## 2. Objectives

The purpose of this double-blind randomized study was to investigate the effects of the combined use of remifentanil used for pain management in patients who planned to undergo ESWL, and the use of EMLA, a topical local anaesthetic.

## 3. Patients and Methods

After the approval of the Ethics Committee of University Faculty of Medicine and the informed consent of patients, a total of 60 ASA I-II patients with renal stone disease between 18-70 years of age who were scheduled to undergo elective ESWL using the Dornier® lithotripsy (Donier MedTech, Germany) were enrolled in the study. The exclusion criteria were patients with opioid allergy, obesity (BMI > 30), methemoglobinemia, liver disease, alcohol consumption, patients using preoperative opioids and derivatives, cardiovascular and neuropsychiatric medications, pregnant and nursing mothers. No preoperative sedative-hypnotic drugs or antiemetic agents were used. Prior to the ESWL procedure, the data recorded for each patient included age, sex, ASA status, and location and diameter of stones. The patients were double-blindedly randomized into two groups: the first group (group E, n = 30) was administered a total of 10 gr of 5% EMLA cream in the thickness of 3-5 mm on a marked area 10 cm by 15 cm; the second group (group P, n = 30) was administered a cream with no analgesic effect in the same amount and quality as a placebo one hour before the ESWL. Prior to the procedure, each patient was asked to score their level of pain from their kidney stones on a VAS and VRS (Verbal Rating Scale 0-3). Standard monitorization included electrocardiography, heart rate, non-invasive arterial blood pressure, respiratory rate and SpO_2_ and all patients were administered O_2_ via a facial mask at a rate of 6 liters/min and Remifentanil PCA with a dose of 10 µgr (patient-controlled analgesia, Abbott) (in a bolus of 10 µg, with a lock-out time of 5 minutes) and the patients were asked to press the button when he or she felt pain. During the procedure, vital parameters and VAS and VRS values were recorded for each patient every 10 minutes. Remifentanil administration was discontinued 3 minutes before the termination of the ESWL procedure and PCA demands and deliveries were recorded for each patient. An unpaired t-test was used to compare demographic data and Bonferroni’s test to compare the pain scores between the two groups.

## 4. Results

**Figure 1.**
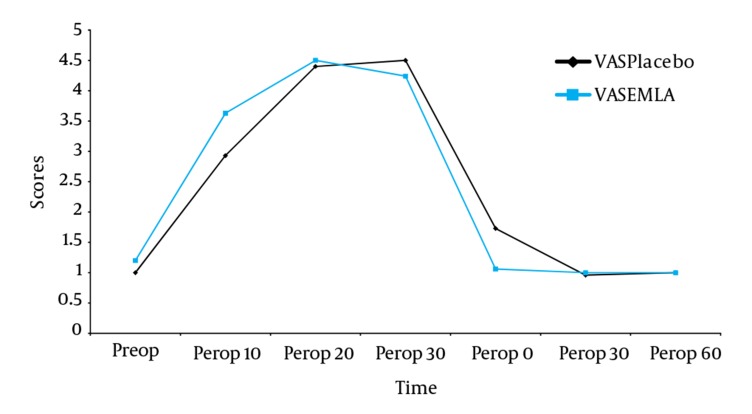
VAS Score Differences Comparing EMLA and Placebo

**Figure 2.**
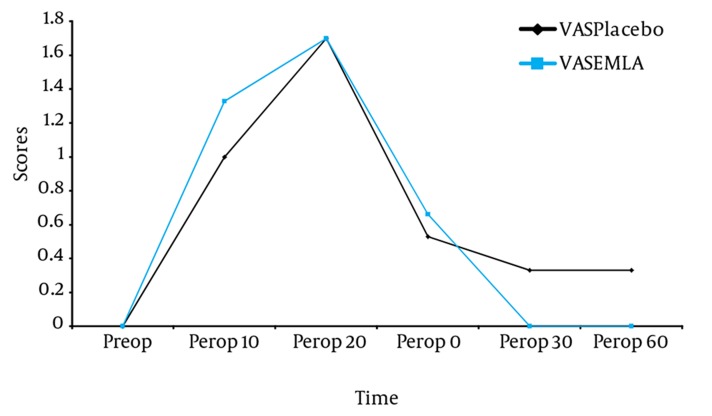
VRS Score Differences Comparing EMLA and Placebo

The results are presented as mean ± standard value and a *P* value of < 0.05 was considered statistically significant. Demographic data, duration of ESWL procedure, location of stone, maximum energy and the number of shock waves were similar between the groups (Table 1). Preoperative, perioperative and postoperative VAS and VRS scores showed similar differences over time (Figures 1 and 2). The remifentanil consumption and the incidence of side effects due to the use of remifentanil were similar between the groups. Both groups had similar number of PCA demands and PCA deliveries (Table 2).

**Table 1.** Demographic Data

	Group E (n = 30)		Group P (n = 30)	*P* value
**Age, y, Mean ± SD**	48.5 ± 2.2	43.4 ± 2.5		0.13
**Male/Female, No.**	17/13	16/14		
**Height, cm, Mean ± SD**	165.6 ± 1,7	167.9 ± 1.6		0.4
**Weight, kg, Mean ± SD**	70.7 ± 2.1	69.5 ± 2.3		0.7
**Duration of The ESWL Procedure, min, Mean ± SD**	26.1 ± 8.1	30.3 ± 8.8		0.06
**Location of Stone:**				
**Renal Pelvic**	9	8		
**Upper calyx**	6	8		
**Middle calyx**	4	2		
**Lower calyx**	8	8		
**Ureter**	3	4		
**Shock Waves, No.**	1988.3	2000		0.46
**maximum energy, mv**	21.1	21		0.32

**Table 2.** Data Concerning Remifentanil and PCA, VAS Pain Scores

	Group E (n = 30)	Group P (n = 30)	*P* value
**Remifentanil dose, µg, Mean ± SD**	17.3 ± 15.5	21.3 ± 12.8	0.29
**Number of PCA demands, Mean ± SD**	5.9 ± 6.3	5.2 ± 8.3	0.79
**Number of PCA deliveries, Mean ± SD**	2.1 ± 1.5	1.7 ± 1.1	0.72
**Preoperative VAS, Mean ± SD**	1.2 ± 0.8	1.0 ± 0	0.16
**Peroperative VAS, Mean ± SD**	3.9 ± 0.9	4.1 ± 0.4	0.84
**Postoperative VAS (0 min), Mean ± SD**	1.1 ± 0.3	1.7 ± 1.7	0.72
**Postoperative VAS (60 min), Mean ± SD**	1.0 ± 0.5	1.0 ± 0.0	1.00
**Postoperative Side effects, yes/no**			
**Hypotension**	0/30	0/30	
**Respiratory depression**	0/30	0/30	
**Nausea and Vomiting**	3/30	3/30	
**Dizziness**	3/30	4/30	

Abbreviations: PCA, Patient controlled analgesia; VAS, Visual Analogue Scale.

## 5. Discussion

ESWL is a commonly used treatment for patients with kidney and uretheric stones, offering a high efficacy and a low complication rate and is performed on an outpatient basis in most centers. ESWL uses acoustic shock waves to break up kidney stones, during which pain at the entry site of shock waves and deep visceral discomfort is experienced (11). For this reason, there are numerous studies using opioids (1, 11, 12). Even though opioids are used extensively because of their high efficiency, their side effects such as bradychardia, hypotension, respiratory depression, sedation, nausea-vomiting, and itching can lengthen their hospital stay which has led clinicians to seek alternatives. Several studies on this issue have attempted to determine various regimens of remifentanil; the optimal bolus dose and infusion rate of remifentanil in itself or compared with other opioids such as sufentanil, alfentanil and fentanyl (11, 12). These studies compared a remifentanil bolus of 10 µg and remifentanil infusions of 0.05 µg/kg/min and 0.1 µg/kg/min and demonstrated that the administration of bolus combined with low dose infusion had a beneficial analgesic effect and a low incidence of side effects (12). In this study, we used remifentanil and patient controlled analgesia combined with remifentanil bolus of 10 µg. Since 1986, various studies have been conducted on the use of local anesthetics for analgesic purposes during treatment (9). Local anesthetics were also shown to be effective in achieving analgesia during ESWL and only 5% of these patients required general anaesthesia (9). There are a number of studies concerning the use of topical EMLA cream for this purpose (9, 13). Even though the skin is where the pain is experienced most intensely as a result of the shock waves during the procedure (6, 14) and EMLA cream is effective in relieving pain, patients usually require additional analgesia since the pain related to ESWL has both cutaneous and visceral components (2, 3). A study by Bierkens et al. (15) reported a 23% lower use of fentanyl and lower pain scores compared to placebo, however, the results did not reach statistical significance. Monk et al. (6) compared EMLA cream with IV fentanyl and reported that even though the application of EMLA cream produced cutaneous analgesia, it failed to produce an opioid-sparing analgesic effect, and was not superior to placebo. They also reported that EMLA cream produced no decrease in postoperative side effects and recovery times. In conclusion, considering EMLA’s slow onset, the inability to identify the precise entrance site for the shock waves and the high cost of the drug, the routine use of EMLA was not recommended by the authors (6). Barcena et al. (16) conducted a study on 20 patients who had been unable to tolerate pain without IV analgesia during ESWL. In this study, 10 gr of EMLA cream was applied on the skin over the area of 64-100 cm ^2^ 60 minutes before the second session. Despite higher voltages, lower pain scores were found in patients for whom EMLA cream was used and only two patients required further analgesia. In addition, all patients required additional fentanyl in the first session without EMLA. In a study by Ganapathy et al (17), one group received 30 gr EMLA cream and the other group received a placebo 60-90 minutes before the procedure. All patients received 5 mcg/kg of alfentanil via a PCA machine with a lockout time of 3 minutes and no significant differences were noted in pain scores, side effects and duration of stay in the post anaesthesia care unit between EMLA cream and placebo. In a double-blind randomized controlled study of 60 ASA I-III patients between 18-70 years of age, aiming at investigating the effect of EMLA cream in lithotripsy by Terri et al. (6), one group received 30 gr of EMLA cream applied to a 15x20 cm area of skin 90 minutes prior the procedure and the other group received placebo with the same appearance and consistency and patients with pain received an additional bolus of alfentanil 5 µg/kg and an infusion of 0.5 µg/kg/min. The dose of alfentanil was doubled in those patients with continuing pain. They also reported that there was no change in pain scores at energy levels of 10,12,15 mV but there was a significant decrease in pain at energy levels of 18 and 20 mV in the EMLA group. However, no significant differences were noted in alfentanil use between the two groups. In the present study, similar to those of Ganapathy and Terri (6, 17), 10 gr of EMLA cream was applied to a 10x15 cm area of skin 1 hour before the procedure. Both authors preferred alfentanil as the opioid. In this study, we preferred remifentanil, which has a short duration. We administered remifentanil at a bolus dose of 10 µg with a lockout time of 5 minutes. As in the study by Ganapathy et al., no basal infusion was administered. Thus, keeping the dose of remifentanil at the lowest tolerable level, we tried to assess how effective EMLA cream was. In this application, no hypotension or respiratory depression due to remifentanil was observed. Side effects such as nausea-vomiting and dizziness were similarly low in both groups. No patients had severe pain necessitating the administration of other analgesics or the termination of the procedure. Even though it has been suggested that topical anesthetics used for the elimination of cutaneous component of pain can provide a more comfortable analgesia by reducing the use of opioids and their side effects, we demonstrated in this study that EMLA cream did not lead to a decrease in the dose of remifentanil compared to a placebo during ESWL. In conclusion, we found that EMLA cream combined with PCA using remifentanil was not significantly superior to a placebo in ESWL and did not lead to a decrease in the dose of remifentanil used during ESWL. However, there are different application schemes for EMLA. We do consider that the investigation of the use of EMLA cream alone or combined with other IV analgesia regimens will be able to give further insight into the efficacy of EMLA cream.
